# Novel copy number variation of the *TGFβ3* gene is associated with *TGFβ3* gene expression and duration of fertility traits in hens

**DOI:** 10.1371/journal.pone.0173696

**Published:** 2017-03-16

**Authors:** Lantao Gu, Chenghao Sun, Yangzhang Gong, Mei Yu, Shijun Li

**Affiliations:** 1 Key Laboratory of Agricultural Animal Genetics, Breeding and Reproduction of Ministry of Education, College of Animal Science and Technology, HuaZhong Agricultural University, Wuhan, HuBei, China; 2 Huadu Yukou Poultry Industry Co. Ltd, Beijing, China; China Agricultural University, CHINA

## Abstract

Improvements in the duration of fertility (DF) could increase the interval between successive artificial inseminations, thereby decreasing the cost associated with production of hatching eggs. The molecular mechanisms involved in DF in hens remains under-explored. In this study, expression levels of the *transforming growth factor-β genes* (*TGFβs*: *TGFβ1*, *TGFβ2*, *TGFβ3*) were investigated in utero-vaginal junctions (UVJs) of hens with long DF (Group L, n = 10) and short DF (Group S, n = 10). *TGFβ1* and *2* tended to exhibit higher expression levels in UVJs from Group L hens. The expression levels of *TGFβ3* mRNA and protein were significantly increased in UVJs of hens from Group L compared to hens in Group S. Consistently, six *TGFβs* downstream genes (*DAXX*, *MEKK1*, *T-BET*, *GATA-3*, *TAK1*, and *FOXP3*) associated with the immune response were found to be significantly differentially expressed in UVJs of Group L than Group S hens. In addition, four SNPs were identified in intron 1 of *TGFβ3*, and these SNPs were significantly associated with DF traits (*P* < 0.05). Furthermore, we identified multi-copy and copy number variants (CNVs) in chicken *TGFβ3* and later determined significant associations between *TGFβ3* CNVs and DF traits in hens. Specifically, *TGFβ3* copy number exhibited a significant positive correlation with its expression (*P* < 0.05). Collectively, our results suggest that chicken DF traits may be regulated by the expression of *TGFβ3* in UVJ. Meanwhile, the copy number variation in the *TGFβ3* gene identified in this study seems to be one marker for DF traits.

## Introduction

In the modern egg production industry, artificial insemination (AI) has been widely used in order to reduce production costs and improved quality of the progeny [1]. However, the labor costs associated with AI are high and increase as the intervals between consecutive inseminations decrease. This interval is determined by duration of fertility (DF) traits that are generally defined as the number of days after a single AI when hens lay fertile eggs [2]. There are two traits most commonly used to observe and reflect the DF: the number of days following insemination until the last fertile egg is produced (DN) and the number of fertile eggs after a single AI (FN) [3–6]. In previous studies, the possibility of increasing the AI intervals by improving DF via selection of these two traits has been proven [7–10]. Moreover, current studies have been done using traditional selection methods. However, few studies report on DF improvement by applying modern molecular markers-assisted selection technology aimed to uncover the differential expression genes of DF traits or reliable DF-associated molecular markers.

The biological basis of DF has been reported to be associated with how hens store a population of sperm in their oviduct for days or weeks after insemination [11–13]. To store sperm, hens possess specialized simple tubular invaginations referred to as sperm storage tubules (SSTs) located in the utero-vaginal junction (UVJ) mucosal folds. Here, the spermatozoa are stored and ultimately released for upward transport toward the infundibulum for ova fertilization [14]. In previous studies, the population of immune cells in the UVJ tissues of infertile hens significantly increased in hens receiving AI [15, 16], and infiltration of lymphocytes into the SSTs of hens with low fertility were observed [17]. Along these lines, Bakset reported that successful sperm storage in the SSTs depends on the immune privilege of the sperms which is thought to relate to an allergen residing in SSTs [18]. Chicken transforming growth factor-β genes (*TGFβs*: *TGFβ1*, *TGFβ2*, *TGFβ3*) belongs to a large family of growth and differentiation factors that play a pivotal role in a great variety of biological activities including morphogenesis, development, and differentiation [19–21]. The genes in this family of growth and differentiation factors have been reported to regulate the expression of many other cytokines while suppressing immune system reactions via inhibition of T-and B-lymphocytes proliferation [22–24]. Moreover, the expression of these genes have also been described to be upregulated in the UVJ following AI [25]. Therefore, Das et al. reported that the immune privilege of sperm at least in part may be realized by increasing local immune suppressing via up-regualting *TGFβs* expression levels in the UVJ [17, 26].

In this study, the *TGFβs* were explored as possible DF-related genes. Since DF traits is highly variable among populations of hens, we hypothesized that relative differential expression and genetic variation of *TGFβs* in UVJ tissues existed. Such clarification is useful to understand and improve DF traits in chicken breeding.

## Results

### Data collection

Two DF traits (DN and FN) of 700 hens were measured in three age periods (62–64, 65–67 and 68–70 weeks). Their characteristics—including phenotypic values (mean ± standard deviation (SD)), the coefficient of variation, repeatability, and phenotypic correlation—are presented in Table 1. Both DN and FN were show high individual variability.

**Table 1:** Characteristics of DF traits (DN and FN) in 700 hens.

Parameters	DN	FN
**Phenotypic values (mean±SD)**	13.34±2.42	9.50±2.00
**Coefficient of variation**	18.13%	20.90%
**Percentiles (2.5%-97.5%)**	6.70–17.00	5.33–13.00
**Repeatability**	0.44	0.42
**Correlation coefficients (FN & DN)**	0.73	

DN = the days post-insemination until last fertile egg

FN = the number of fertile eggs after a AI

### Expression patterns of *TGFβs* in UVJs of hens from long and short DF groups

**Fig 1:**
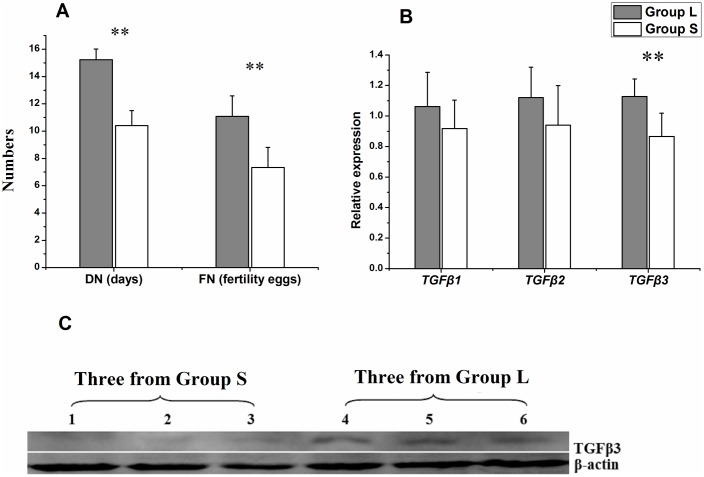
Comparison of *TGFβs* expression levels in UVJ tissue. (A) Quantification of DF traits (DN = the days post-insemination until last fertile egg; FN = the number of fertile eggs after AI) of group L and group S hens are shown. (B) The levels of *TGFβs* mRNA expression in UVJ tissues of group L and group S. Data in (A) and (B) are presented as the mean ± SD for each group (n = 10), **P < 0.01. (C) The levels of TGFβ3 protein expression in UVJ tissues of three hens with short DF traits from Group S (1, 2 and 3) and three hens with long DF traits from Group L (4, 5, and 6).

*TGFβs* (*TGFβ1*, *TGFβ2*, *TGFβ3)* mRNA expression level in UVJ of hens from the long DF group (Group L, n = 10) and short DF group (Group S, n = 10) were tested. The results showed that the *TGFβs* mRNA expression level of Group L hens tended to be higher than that of Group S hens, especially for *TGFβ3*, its expression levels were significantly higher in UVJs from Group L hens as compared to those in Group S (*P <* 0.01). Therefore, the TGFβ3 protein expression levels in UVJs were further tested using Western-blot analysis. Result showed that the TGFβ3 protein expression levels of hens with long DF traits from Group L was also higher than that with short DF traits from Group S (Fig 1).

### Expression patterns of downstream genes of *TGFβ3* in UVJs of hens from long and short DF groups

**Fig 2:**
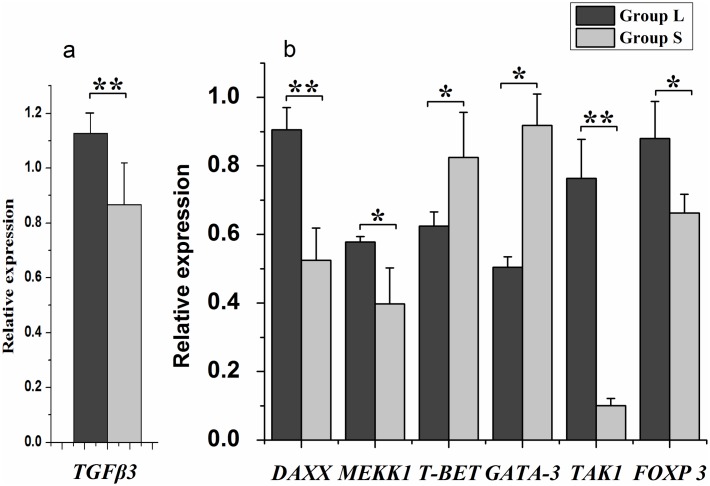
Expression levels of 6 *TGFβs* downstream genes in UVJ. **(a)** Group L and Group S (n = 10 for each group) hens exhibit differential expression of *TGFβ3* in UVJ. **(b)** Differential expression of 6 *TGFβ3* downstream genes in UVJ for the two groups is shown. *P < 0.05, **P < 0.01.

Additionally, the expression levels of 6 *TGFβ3* downstream genes in UVJ tissues were also found to be significantly different between Group L and Group S hens (Fig 2).

### SNPs identification and its association analysis with DF traits

**Fig 3:**
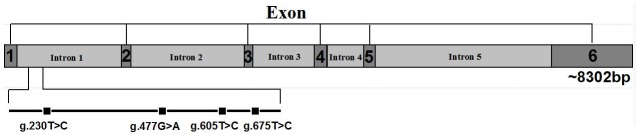
Chicken *TGFβ3 g*enetic structure (NC_00692.3). Four SNPs were identified on intron 1 of the chicken *TGFβ3* gene and named g.230T>C, g.477A>G, g.605T>C, and g.675T>C.

A total of four SNPs were identified on intron 1 of the chicken *TGFβ3* gene through PCR sequencing, named g.230T>C, g.477A>G, g.605T>C, and g.675T>C (Fig 3). The four SNPs were genotyped in a population of 652 hens and found to be significantly associated with DN and FN (P < 0.05) with the exception of the SNPs A477G and T607C on DN (Table 2).

**Table 2:** Four SNPs of *TGFβ3* and their associations with DF traits in hens.

**Traits**	**g.230T>C**			
	**CC (33)**	**TC (448)**	**TT (171)**	**P values**
**DN(days)**	12.95±0.42^**AB**^	13.53±0.11^**A**^	12.93±0.18^**B**^	0.015*
**FN(numbers)**	9.64±0.35^**AB**^	9.68±0.09^**A**^	9.09±0.15^**B**^	0.004**
	**g.477A>G**			
	**AG (300)**	**GG (352)**	**P values**	
**DN(days)**	13.50±0.14	13.20±0.13	0.13	
**FN(numbers)**	9.69±0.12^**a**^	9.38±0.11^**b**^	0.049*	
	**g.605T>C**			
	**CC (62)**	**TC (300)**	**TT (290)**	**P values**
**DN(days)**	12.85±0.31	13.50±0.14	13.28±0.14	0.079
**FN(numbers)**	8.99±0.25^**a**^	9.69±0.12^**b**^	9.46±0.12^**ab**^	0.024*
	**g.675T>C**			
	**CC (109)**	**TC (316)**	**TT (227)**	**P values**
**DN(days)**	12.98±0.23^**B**^	13.70±0.14^**A**^	13.02±0.16^**B**^	0.001**
**FN(numbers)**	9.14±0.19^**B**^	9.83±0.11^**A**^	9.28±0.13^**B**^	0.001**

DN = days post-insemination until last fertile egg; FN = the number of fertile eggs after AI. Among genotypes within each SNP for each trait, values without common capital letters indicate extremely significant differences (P < 0.01); values without common lower-case letters indicate significant differences (P < 0.05).

*P < 0.05;

**P < 0.01.

### Identification of multi-copy in *TGFβ3* of hens

**Fig 4:**
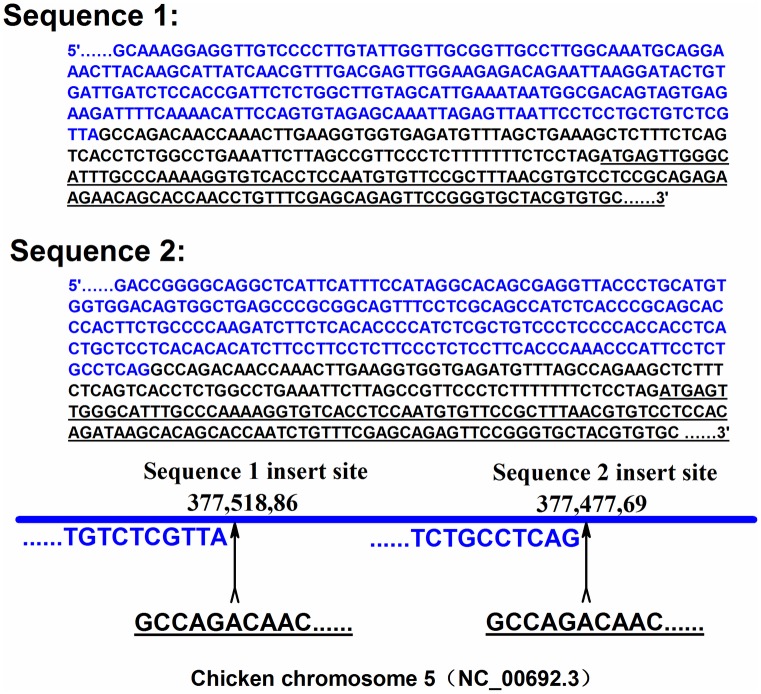
Two *TGFβ3* gene 5' flanking region sequences (sequence 1 and sequence 2) and their location on chicken chromosome 5 (NC_00692.3) were identified. The differing region between these two sequences is marked with blue font and the similar region is marked with black font. In the similar region is the *TGFβ3* DNA sequence, marked by underlining. Blast result of blast shows that the two sequences are located at two sites in the chromosome 5.

We carried out a DNA walking experiment. Two *TGFβ3* 5' flanking region sequences (Sequence 1 and Sequence 2) were identified, as show in Fig 4. These results implied that there are non-allelic copy of *TGFβ3* gene that existed in the chicken genome. Therefore, the two sequence were blasted with the reference sequence, and the two sequences were aligned at two site on chicken chromosome 5 (NC_00692.3).

**Fig 5:**
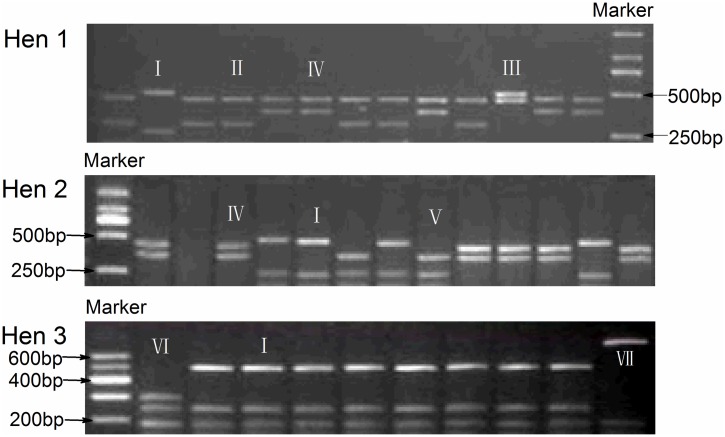
PCR-RFLP results of monoclonal colonies. Each monoclonal colony contains a allelic fragment of *TGFβ3* DNA. Seven different restriction patterns are marked from **I to VII**.

**Fig 6:**
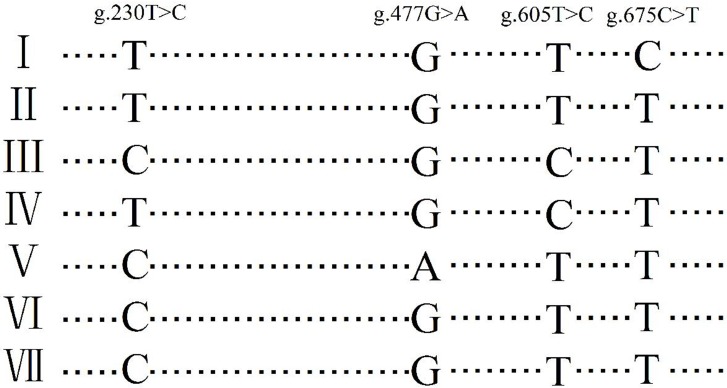
Sequencing results of 7 monoclonal colonies (I, II, III, IV, V, VI and VII). The 7 monoclonal colonies respectively carried 7 kinds of allelic fragments of *TGFβ3* DNA which differed by haplotypes of 4 SNPs.

In addition, the *TGFβ3* DNA fragment which contains the 4 SNPs identified above were amplified and sequenced using DNA samples of 3 hens (1, 2 and 3). All of the 4 SNPs affect the restriction site of BsaJ I. The PCR-RFLP results of their monoclonal colonies revealed seven different restriction patterns (Fig 5). Sequence analysis demonstrated that the seven monoclonal colonies respectively carried seven types of *TGFβ3* DNA fragments which had differing haplotypes of the four SNPs (Fig 6). Interestingly, as shown in Fig 3, in certain cases, more than two types of restriction patterns were expressed in an individual; for example hens 1 and 3 each had three and hen 2 had 4. This finding means that in the genome of these three hens, there are at least three or more types of allelic fragments for *TGFβ3* DNA.

In a diploid genome, there are generally two allelic copies of genes. However, the above results showed that there are non-allelic copies and more than two copies of *TGFβ3* DNA fragments in the chicken diploid genome. A reasonable interpretation of these data is that *TGFβ3* may be a multi-copy gene.

### Copy number variation of *TGFβ3* and its association analysis with DF traits in hens

**Fig 7:**
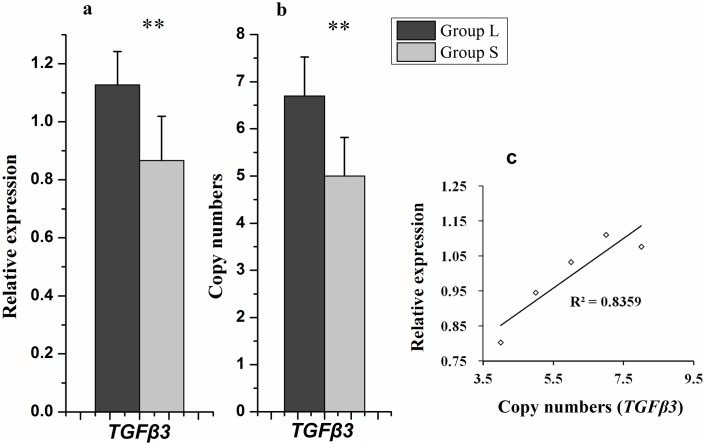
Relationship of *TGFβ3* copy number and its expression in UVJ. **(a)** Difference in *TGFβ3* expression levels between Group L and Group S. **(b)** Comparison of *TGFβ3* copy numbers between Group L and Group S. Data are presented as the mean ± SD of each group (n = 10). **(c)** Correlation between *TGFβ3* expression level and its copy numbers is shown. **P < 0.01.

Multi-copy genes always show copy number variation. Thus, using the qPCR method, copy number variation of *TGFβ3* in 151 hens were evaluated. The results showed that copy number varied from 4 to 8 and significantly associated with DF traits in hens (*P* < 0.05). The hens with long DF traits have more copy numbers than hens with short DF traits (Table 3). Additionally, the copy numbers of hens in Group L and Group S was compared and correlated with *TGFβ3* expression levels in the UVJ. The result showed that the *TGFβ3* copy number of Group L was significantly higher than that of Group S (Fig 7) and howed a significant positive correlation with *TGFβ3* expression (*P* < 0.05).

**Table 3:** Copy number of *TGFβ3* and its association with DF traits in hens.

Copy number	DM	FN	N
**4**	10.82±3.09^a^	7.63±2.36^a^	13
**5**	11.4±3.26^a^	8.15±2.73^a^	36
**6**	12.07±3.11^a^^b^	8.66±2.70^a^	50
**7**	13.34±3.83^b^	9.91±2.93^b^	36
**8**	13.92±2.68^b^	10.48±2.29^b^	16
**P-value**	0.014*	0.003**	

DN = the days post-insemination until last fertile egg; FN = the number of fertile eggs after AI.

^a, b^ Means within a column for each trait lacking a common superscript differ (*P <* 0.05).

*P < 0.05;

**P < 0.01.

## Materials and methods

Ethics Statement: This study was carried out in strict accordance with the recommendations in the Guide for the Care and Use of Laboratory Animals of the Standing Committee of Hubei People's Congress (No. 5) and approved by the Biological Studies Animal Care Committee of Hubei Province, P.R. China, and the Ethics Committee of Huazhong Agricultural University, P. R. China. All surgery was performed under sodium pentobarbital anesthesia, and all efforts were made to minimize suffering.

### Hens management, data collection and DNA isolation

A total of 700 healthy Jing Hong hens (30 weeks old) were obtained from the poultry farm of Huadu Yukou Poultry Industry Co. Ltd, Beijing, China. All birds were raised in individual cages, kept in identical light/dark cycles and had ad libitum access to water from 25 weeks to the end of the experiment.

All hens were artificially inseminated once with 2.00×10^8^ sperms issued from pooled ejaculates collected from 20 Jing Hong rooster stocks. To prevent any undesirable effects of the interval between inseminations and oviposition on subsequent fertility, inseminations was done in the afternoon on the first day of 28, 31 and 34 weeks. Eggs were collected and marked daily from day 2 to day 22 after AI.

Fertility was checked by candling eggs on 10 days of incubation (dead embryos were considered as fertile). DF data were expressed in terms of DN (the number of days post-insemination until last fertile egg) and FN (the number of fertile eggs after a single AI).

Blood samples of the 700 hens were collected and DNA was extracted using the phenol-chloroform method. Concentration of each DNA sample was quantified by spectrophotometer ND-2000 (Nano-Drop, USA) and adjusted to be approximately 50 ng/μL.

### Real-time PCR and Western blot analysis of *TGFβs* and the downstream genes of *TGFβ3* in UVJ of hens

Upon completion of the above experiment, a total of 20 hens were separated from the experimental population and assigned to Group L (long DF group, n = 10) or Group S (short DF group, n = 10) according to their DN and FN traits. On the 13^th^ day after AI, the hens were euthanized by decapitation under anesthesia. UVJ tissues were dissected immediately and adhering connective tissues were removed [14]. The UVJ mucosa was cut into small pieces and frozen at -80°C prior to use.

Total RNA was isolated using Trizol reagent (Invitrogen, Foster City, CA, USA), following the recommended manufacturers protocol. The quality and quantity of RNA samples were detected by 1.0% agarose gel electrophoresis and absorbance optical density (OD) at a 260/280 nm ratio, respectively. For cDNA synthesis, 1.5 μg of total RNA underwent reverse transcription using EasyScript^™^ one-step gDNA Removal and cDNA Synthesis SurperMix (TansGen Biotech, Beijing).

In total, expression levels of nine genes were investigated. The nine genes included three *TGFβs* and six downstream genes of *TGFβ3*, which were death domain associated protein (*DAXX*), mitogen-activated protein kinase kinase kinase 1(*MEKK1*), T-box 21(*T-BET*), GATA binding protein 3(*GATA-3*), TGF-beta activated kinase 1(*TAK1*) and Pseudopodoces humilis forkhead box P3 (*FOXP3*).

The relative quantity of *TGFβs* mRNA was detected using Quantifast^™^ SYBR Green PCP Kit (QIAGEN, Germany) under the following conditions: 94°C for 5 min, followed by 40 cycles of 20 s at 94°C, 20 s at 60°C, and 20 s at 72°C. Each sample was assayed in triplicate, and chicken *GAPDH* was used as reference. Fold-change values were calculated using the comparative 2^−ΔΔCt^ (ΔΔCt = ΔCt _target sample_ − ΔCt _control sample_) method.

In the Western-blot experiments, UVJ tissue protein lysates were generated using the Tissue Protein Extraction Reagent (Thermo Scientific, USA). Each sample of 30 μg total protein was separated by SDS-PAGE (12% separating gel and 5% stacking gel). After SDS-PAGE, the samples were electrophoretically transferred onto a nitrocellulose membrane (Bio-Rad, USA) and incubated with sheep anti-rabit *TGFβ3* polyclonal antibody (Abcam, USA; 1:1000 dilution) overnight at 4°C. Following the incubation with biotinylated anti-sheep IgG (Abcam, USA; 1:3000 dilution) for 2h, bands were visualized by incubating in chemiluminescent detection reagents to detect protein expression. Bands of *TGFβ3* are measured at approximately 47.5 kDa.

### SNPs identification and genotyping

The PCR sequencing method was used to identify *TGFβ3* SNPs. Briefly, DNA samples of Group L and Group S animals were pooled and amplified using *TGFβ3* specific PCR primers. The PCR products were then gel purified and sequenced by Sangon Bioteh Co. Ltd, Guangzhou, China. The obtained sequences were aligned to screen SNPs based on their differences.

PCR-restriction fragment length polymorphism (PCR-RFLP) technology was used to test SNP genotypes. PCR products were digested by restriction enzymes following the manufacturer’s instructions, and then detected through agarose gel electrophoresis.

### Multi-copy identification and copy number quantitation of *TGFβ3*

Three experiments, including DNA walking, clone-based sequencing and qPCR, were performed in this section:

First, according to DNA walking technology, 3 specific primers were designed based on the *TGFβ3* gene’s 5' flanking region sequence. Then, the DNA samples of 20 hens were pooled and a triplex nested PCR was performed using a Genome Walking Kit (TaKaRa, Japan). The third PCR products were transformed into E coli competent cells, coated onto plates. Monoclonal colonies was selected and sequenced.

Second, the clone-based haplotyping technology, which determine the phase of SNPs on a chromosome based on separating the allelic chromosomes by clone and sequencing, was used for reference [27]. we designed a pair of primer that amplify the *TGFβ3* DNA fragment that contains the 4 SNPs identified above. DNA samples of 3 hens (1, 2 and 3) were amplified individually. PCR products were gel-purified, ligated with PEASY-T1 vector (TransGen Biotech, BeiJing China), and transformed into E coli competent cells and coated onto plates. In this way, amplified *TGFβ3* DNA fragments were separately isolated into monoclonal colonies. Thereafter, the monoclonal colonies are selected and screened by PCR-RFLP using the restriction enzyme *BsaJ I*, which recognized all the 4 SNPs. The monoclonal colonies with different restriction patterns were then sequenced. The obtained sequences was characterized by their base differences at the 4 SNPs locations, and the kinds of sequences the three hens were identified.

Third, quantitative PCR (qPCR) was performed to meansure CNVs [28]. Briefly, two pairs of quantitative primer were designed based on *TGFβ3* (Gene ID: 396438, NC_006092.3) and *PCCA* gene sequences (Gene ID: 418774, NC_006088.3). DNA samples of 160 hens were subjected to a qPCR array using the Quantifast^™^ SYBR Green PCP Kit. Each sample was tested in triplicate. Meanwhile, the quantitative primer amplification efficiency (E) was calculated according to standard plasmid's amplification curves. The copy numbers of *TGFβ3* gene were estimated as Formula (1) and rounded to the nearest whole number.

$$Copy\;number\;of\;TGF\beta 3\;=2\times \;\frac{{\left(1+E_{quantitative\;primer\;for\;TGF\beta 3}\right)}^{Ct\;value\;of\;TGF\beta 3}}{{\left(1+E_{quantitative\;primer\;for\;PCCA}\right)}^{Ct\;value\;of\;PCCA}}$$ (1)

### Statistical analysis

All values are presented as the mean ± standard deviation (SD). The threshold for significance was set at P < 0.05 and for high significance at P < 0.01. Association analyses of SNPs with DF traits were performed using the General Linear Models Procedures (GLM) procedures of SPSS statistical 19 software package, and the genetic effects were analyzed by a mixed procedure according to the following model:

Y=μ+G+e

Where Y = the trait’s phenotypic values; μ = the overall population mean; G = the fixed effect of genotype; e = the random residuals.

## Discussion

In this study, the duration of fertility traits, as well as traits’ regulatory genes and molecular markers, were explored.

The results of DF traits presented here is similar to those studies performed on other egg-type lines, in which DN and FN are both medium repetition rate traits and show high individual variability among hens [29, 30]. Thererfore, relative genetic variability was expected to existed. Similarly, several studies have reported that the biological basis of DF is related to sperm storage, and sperm storage is dependent on sperm immune privilege. This mechanism maybe realized suppressing local immune function by up-regulating *TGFβs* expression in the UVJ [26]. Presence of *TGFβs* increased immune tolerance for anti-sperm immunity in the VUJ, and insufficient expression of *TGFβs* may cause an ineffective suppression of anti-sperm immune responses in the UVJ, decreasing sperm storage and duration of fertility. Thus, Thus, we speculate that DF traits were regulated by the expression of *TGFβs* in the UVJ. Therefore, the expression levels of *TGFβs* was tested in the UVJ. Results showed that the expression levels of *TGFβs* in hens with long DF tended to be higher than that of hens with short DF. *TGFβs* induce the expression of the transcription factor FOXP3 while blocking the expression of GATA-3 and T-BET, thus facilitating the generation of regulatory T cells while decreasing the differentiation of T helper type 1 and 2 cells (Th1 and Th2) [31]. Our data were consistent with this expression pattern: when *TGFβs* were expressed highly in the UVJ of hens with long DF traits, the expression of GATA-3 and T-BET gene were down-regulated while FOXP3 was up-regulated. Additionally, 3 other genes, which are mediated by *TGFβs* in the MAPK singling pathway, also show significant differential expression in the UVJs of hens with different DF traits. These results suggested that chicken DF traits were regulated by the expression of *TGFβs* in the UVJ.

Among the three *TGFβ* family members, *TGFβ3* exhibited the most pronounced significant differential expression differences between hens with both long and short DF traits (*P* < 0.01). With the aim of identifying DF-associated molecular markers, we explored two major gene mutation forms on the *TGFβ3* gene: single-nucleotide polymorphisms (SNPs) and copy number variation (CNV). Four SNPs of the *TGFβ3* gene were identified to be associated with DF traits(*P*<0.05). Moreover, we found two kinds of 5' flanking regions of the *TGFβ3* gene sequence in two non-allelic sites of the chicken genome, as well as more than two haplotypes or copies of *TGFβ3* DNA allelic fragments in the diploid genome of a single chicken. Therefore, multi-copy of *TGFβ3* was believed to exist in chicken genome. Meanwhile, the copy number of *TGFβ3* was found to be highly variable in our population of hens. Association analysis of *TGFβ3* copy number with DF traits showed that the copy number of *TGFβ3* was significant associated with DF traits in hens (*P* < 0.05), hens with long DF trais seems to possess more copy number of *TGFβ3*. Additionally, the biological functions of copy number variation have been reported to be associated with gene expression, and multi-copy of genes has been suggested to modulate its transcription [32]. The *TGFβ3* copy numbers positive correlated with its expression, *while* high *TGFβ3* expression (Group L) hens had much more copy numbers of *TGFβ3* than low expression (Group S) hens. These results suggested that the copy number variation of *TGFβ3* might be a molecular mechanism responsible for its differential expression and may be applied in selecting molecular markers for DF traits. Increasing the copy number of *TGFβ3* in hens using molecular breeding technology may thus extend the hens’ duration of fertility.

Interestingly, both of SNPs and copy number variation of *TGFβ3* were determined to be associated with DF traits in this study. In practice, because of copy number variation, we can not accurately know the allele genetype frequency of SNPs of most diploid genes. Theoretically, it is possible for hens with higher copy numbers to possess different allele genes but be genotyped to be heterozygous in the PCR-RFLP analysis. These analyses indicated that the population of hens with high heterozygosity seems to possess more copy number, which may be the reason for their higher heterozygous genotype frequency than that of homozygous genotypes observed in the four SNPs. Hens with heterozygous genotypes showed longer DF than hens with homozygous genotypes in this study. Therefore, the reliable mutation associated with DF traits actually may indeed be the CNV of the *TGFβ3* gene.

To the best of our knowledge, the first evidence of multi-copy and copy number variation of the *TGFβ3* gene and its association with DF traits. may help deepen our understanding of the function of the *TGFβ3* gene as a marker for genetically improving DF in hens.

Collectively, herein we found that the differential expression of *TGFβ3* gene may be a mechanism respond for individual variability in DF traits. Meanwhile, a novel mutation of multi-copy and copy number variation at *TGFβ3* was identified. Moreover, the copy number variation of *TGFβ3* seems to be a pivotal mutation involving its expression and reliable marker for DF traits in hens.

To the best of our knowledge, this report presents the first evidence of multi-copy and copy number variation of the *TGFβ3* gene and its association with gene expression and DF traits. These results may help deepen our understanding of the function of the *TGFβ3* gene as a marker for genetically improving DF in hens.

## Supporting information

S1 Table Primer used in this study. (DOCX)
