# Supplementary material for: Novel copy number variation of the TGFβ3 gene is associated with TGFβ3 gene expression and duration of fertility traits in hens
Source: PLoS One. 2017 Mar 16;12(3):e0173696. doi: 10.1371/journal.pone.0173696 (PMC5354388; doi:10.1371/journal.pone.0173696)
Supplement: S1 Table — (DOCX) [file pone.0173696.s001.docx]

**S1 Table. Primer used in this study**

| Primer name | Primer sequence (5’-3’) | Product size (bp) | Primers purpose |
| --- | --- | --- | --- |
| TGFβ1-EF | TACATCTGGAGCGCCGACAC | 142 | Expression profile |
| TGFβ1-ER | GCACATTCCGGCCCACGTAG |  |  |
| TGFβ2-EF | TGTTACCCTCCTACAGACTT | 150 |  |
| TGFβ2-ER | TCCATTTCCAGCCAAGATCC |  |  |
| TGFβ3-EF | GCAGTGAGCAGCGCATCGAG | 207 |  |
| TGFβ3-EF | AAGTATGGCAAGGGCAGTGT |  |  |
| DAXX-EF | ACCCCAAAAGCGGAGGAGGAG | 77 |  |
| DAXX-ER | TTGACGCTGGTGGAACTGATG |  |  |
| MEKK1-EF | CAAAACTGCCCAATGGTGTC | 157 |  |
| MEKK1-ER | AGCCATCCTGCAAAATCCAC |  |  |
| T-BET-EF | GGGAACCGCCTCTACCTG | 173 |  |
| T-BET-ER | AGTGATGTCGGCGTTCTGG |  |  |
| GATA-3-EF | TCAGCCACATTTCACCCTTC | 157 |  |
| GATA-3-RF | AGACCGTAAGCATTCAGCAG |  |  |
| TAK1-EF | ATGTGCTCTACTCGATGCTT | 187 |  |
| TAK1-RF | CAAAACTGCCCAATGGTGTC |  |  |
| FOXP3-EF | CTGGAGTATTACCGGCTCAGCAC | 167 |  |
| FOXP3-RF | TTCTTCCAGGTGCGGGTGTT |  |  |
| GAPDH-EF | GGGAAGCTGTGGAGAGATGG | 191 | Expression internal control |
| GAPDH-ER | TCATACTTGGCTGGTTTCTC |  |  |
| TGFβ3-R1F | TTCCGCTTTAACGTGTCCTC | 1537 | Re-sequence |
| TGFβ3-R1R | GCGCTGCTTTGCTATATGCT |  |  |
| TGFβ3-R2F | TCTCTACCGCTCCTTCTATC | 1505 |  |
| TGFβ3-R2R | AGAAAGGAGAATAGGAAGCA |  |  |
| TGFβ3-R3F | ACTTTTCTCACCTCCTCACT | 1395 |  |
| TGFβ3-R3R | CACCGCACTTTCCCACCTCA |  |  |
| TGFβ3-R4F | ATGCCTGAGGTGGGAAAGTG | 1477 |  |
| TGFβ3-R4R | CATGTCTGGCTTGCTACCTT |  |  |
| TGFβ3-R5F | AGACAGTTCATTCCTAAGCA | 1423 |  |
| TGFβ3-R5R | CCTGATGTGACTAATATTCT |  |  |
| TGFβ3-R6F | TCTTCCACCCTTAGGCATCA | 1289 |  |
| TGFβ3-R6R | GAGATCACAAACCACAACAG |  |  |
| TGFβ3-PF | TTCCGCTTTAACGTGTCCTC | 1537 | Polymorphism |
| TGFβ3-PR | GCGCTGCTTTGCTATATGCT |  |  |
| TGFB3-SP1 | \| TCTGGCCTGAGGCAGAGGAA \| \| --- \| |  | DNA walking |
| TGFB3-SP2 | TCACTGCGCTTGGAGCTCGG |  |  |
| TGFB3-SP3 | GGCACACGTAGCACCCGGAA |  |  |

All primer were designed based on the red jungle fowl (Gallus gallus) genome sequence ([GCF_000002315.3](http://www.ncbi.nlm.nih.gov/assembly/GCF_000002315.3/))
